# Supplementary figures and images for: Overview of Gene Expression Dynamics during Human Oogenesis/Folliculogenesis
Source: Int J Mol Sci. 2023 Dec 19;25(1):33. doi: 10.3390/ijms25010033 (PMC10778858; doi:10.3390/ijms25010033)

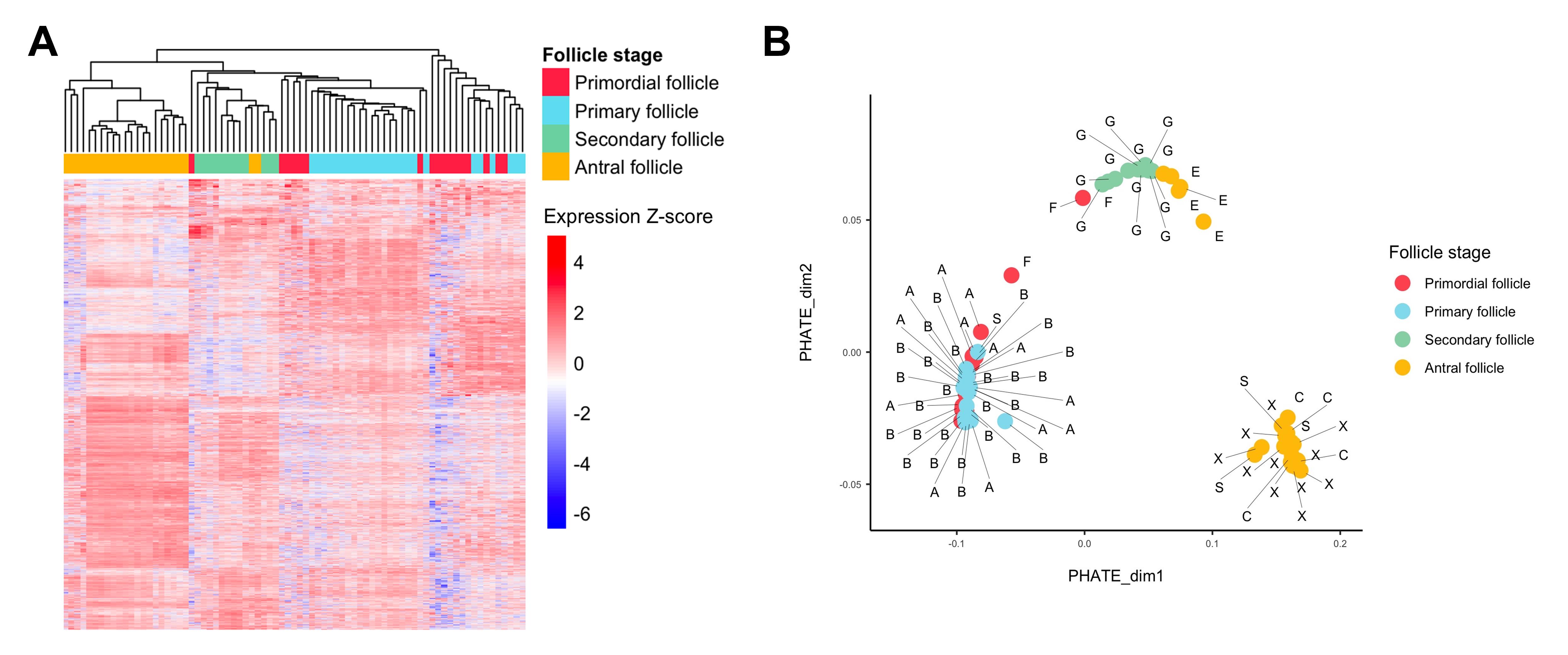

Supplement: Supplementary file 1 [file ijms-25-00033-s001.zip › Supplementary Figure S1.jpg]

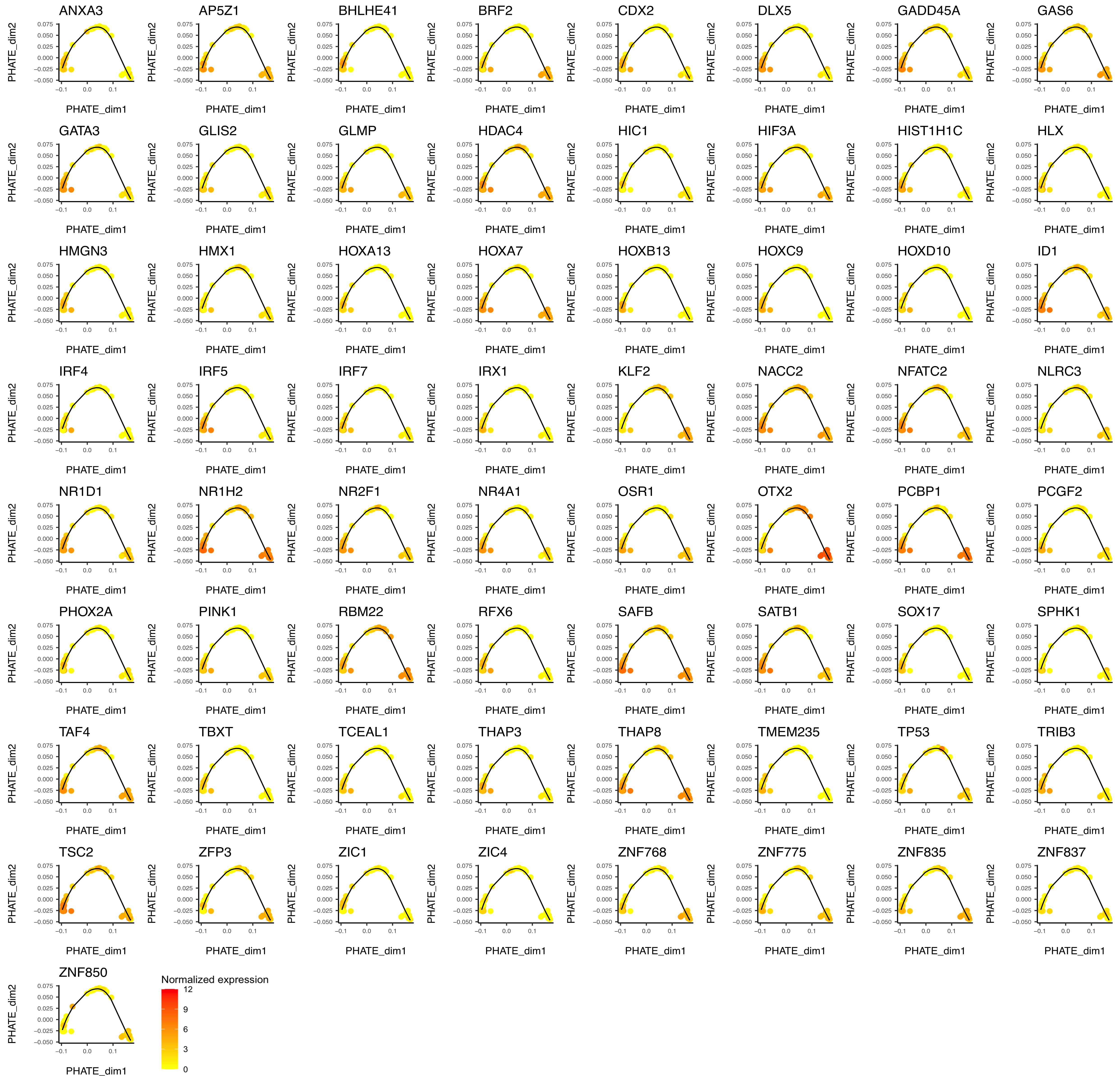

Supplement: Supplementary file 1 [file ijms-25-00033-s001.zip › Supplementary Figure S2.jpg]

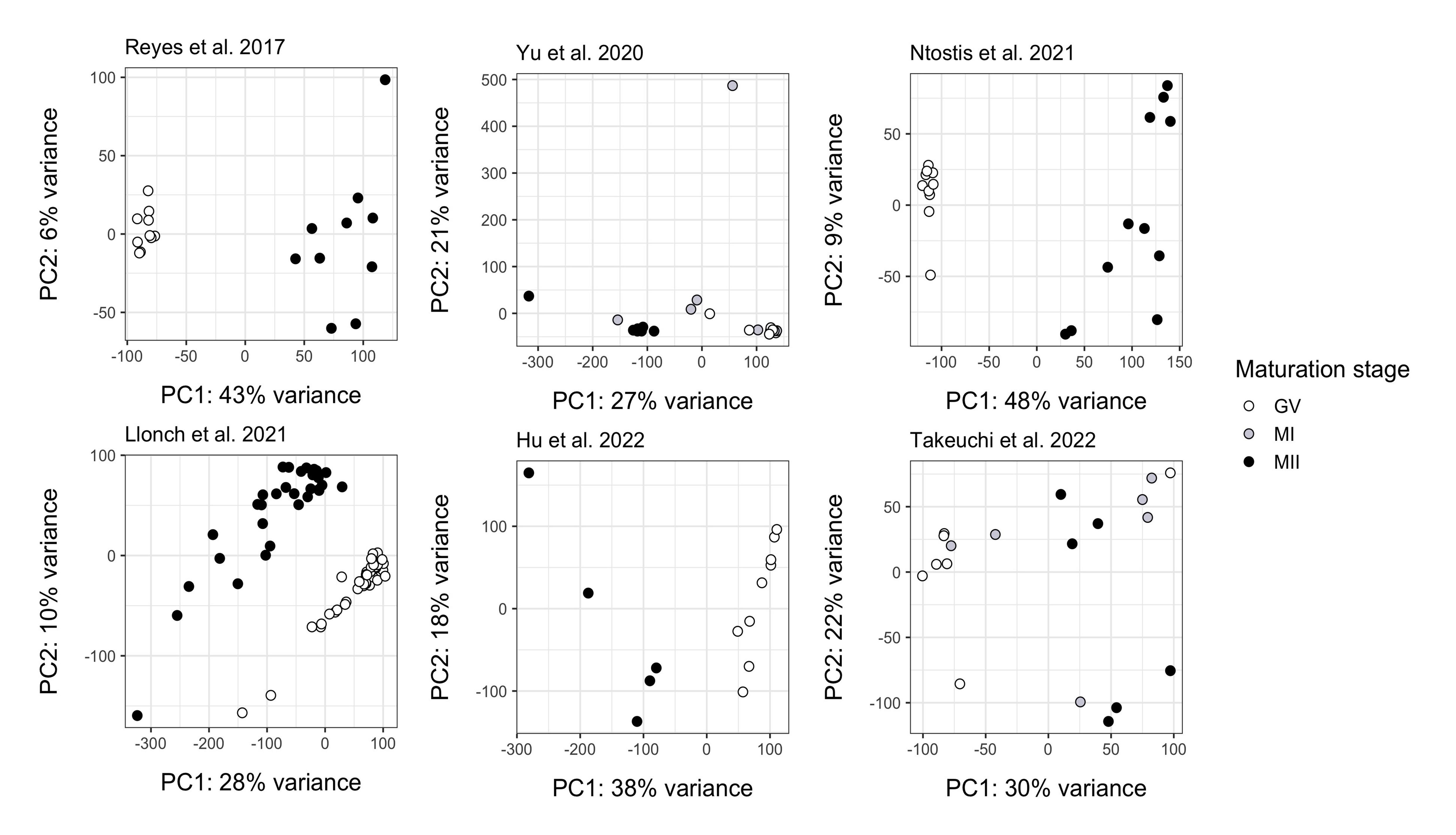

Supplement: Supplementary file 1 [file ijms-25-00033-s001.zip › Supplementary Figure S3.jpg]

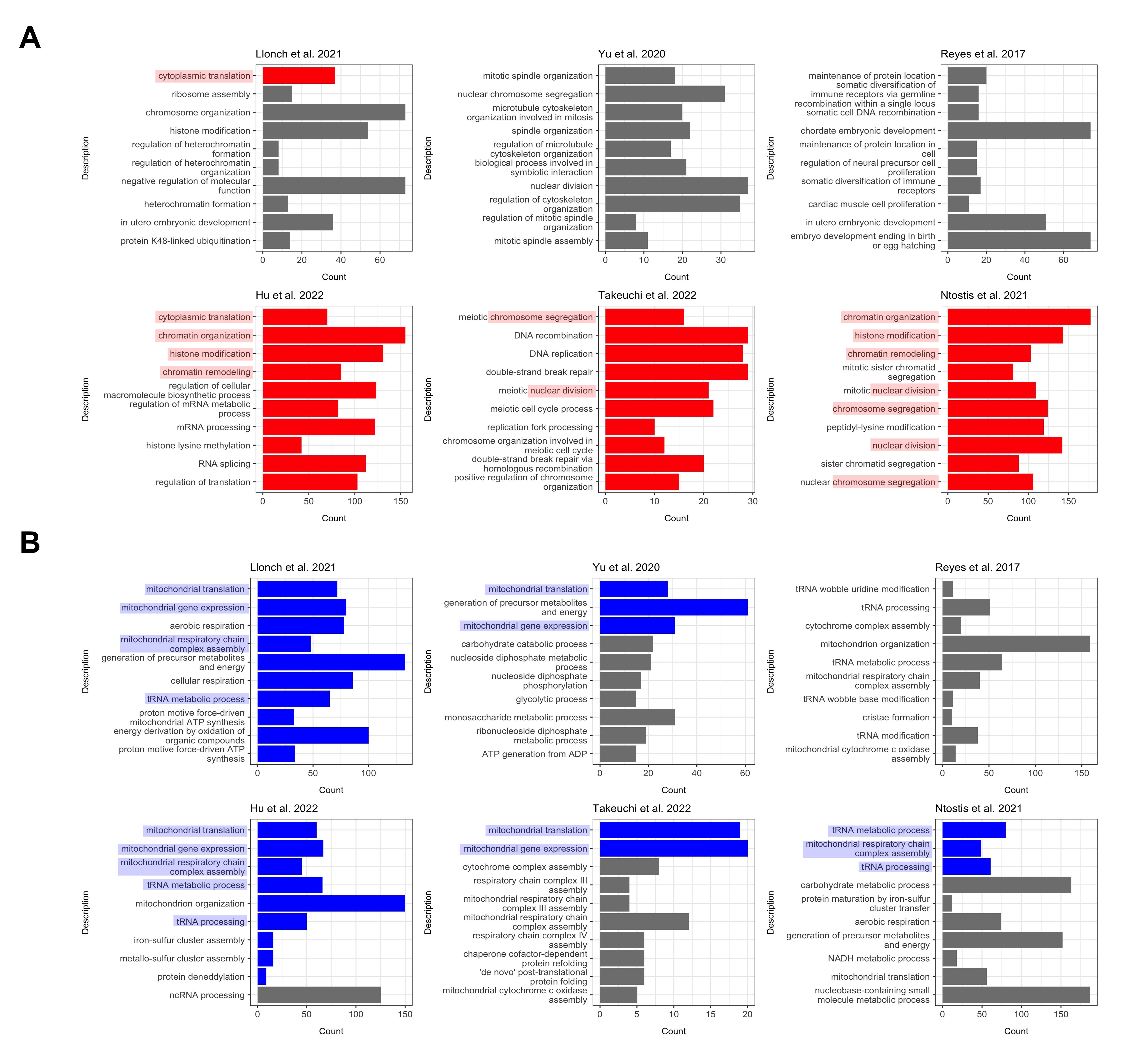

Supplement: Supplementary file 1 [file ijms-25-00033-s001.zip › Supplementary Figure S4.jpg]

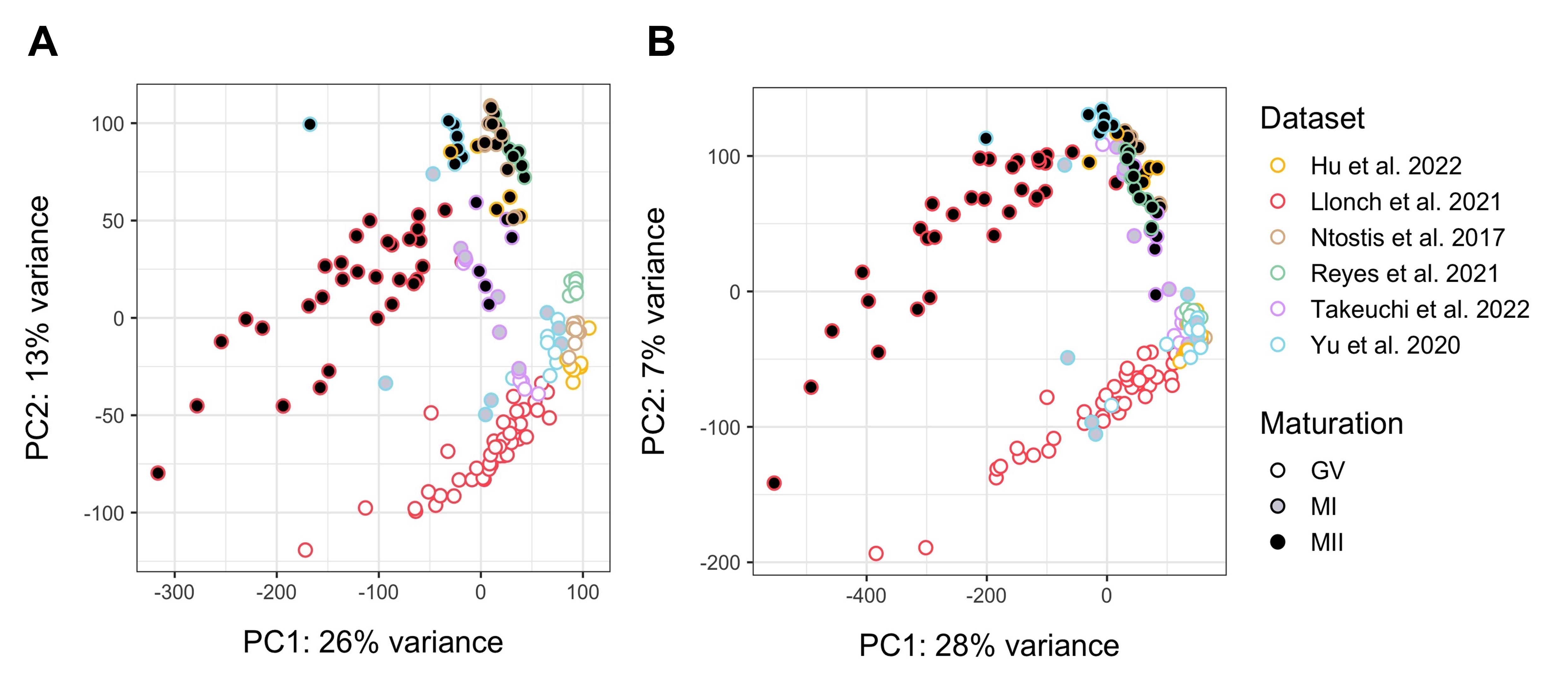

Supplement: Supplementary file 1 [file ijms-25-00033-s001.zip › Supplementary Figure S5.jpg]

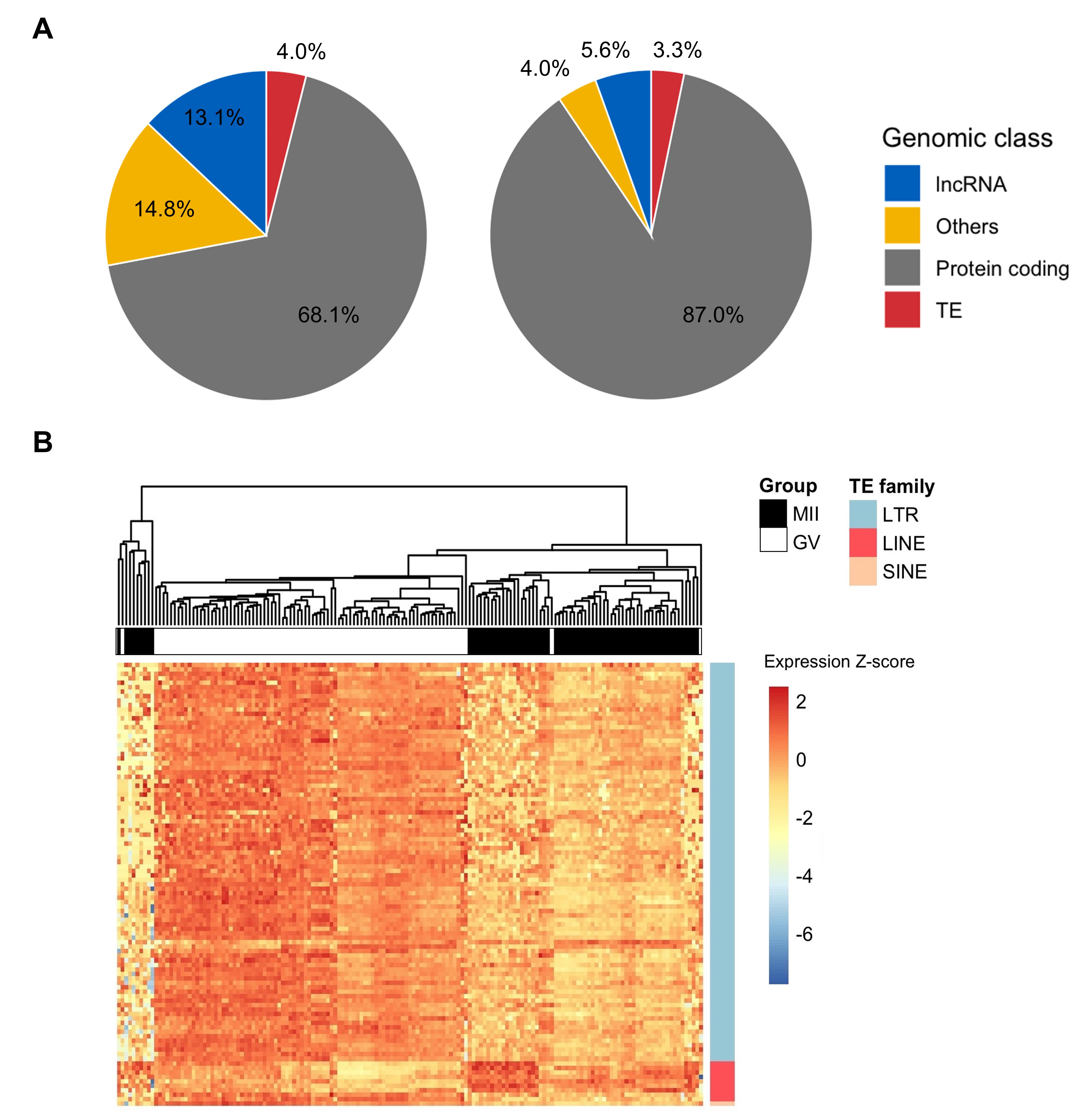

Supplement: Supplementary file 1 [file ijms-25-00033-s001.zip › Supplementary Figure S6.jpg]
